# DDX19A promotes gastric cancer cell proliferation and migration by activating the PI3K/AKT pathway

**DOI:** 10.1186/s12935-024-03448-5

**Published:** 2024-08-03

**Authors:** Yu Cheng, Yanjie Lu, Jing Xue, Xuemei Wang, Lili Zhou, Yu Luo, Yuhong Li

**Affiliations:** 1 Department of Pathology, Chengde Medical College, Chengde, Hebei Province China; 2 Cancer Research Laboratory, Chengde Medical College, Chengde, Hebei Province China; 3 Morphological Experimental Center, Chengde Medical College, Chengde, Hebei Province China; 4 Department of Pathology, Cancer Research Laboratory, Chengde Medical College, Anyuan Road, Chengde, Hebei 067000 China

**Keywords:** Gastric cancer, DDX19A, PIK3CA, PI3K/AKT, Nuclear export

## Abstract

**Background:**

DEAD-box RNA helicase 19 A (DDX19A) is overexpressed in cervical squamous cell carcinoma. However, its role in gastric cancer remains unclear. The present study aimed to explore the role and underlying mechanism of DDX19A in the development of gastric cancer.

**Methods:**

The expression of DDX19A in gastric cancer and paracancerous tissues was evaluated through quantitative polymerase chain reaction, western blotting, and immunohistochemical staining. The biological functions of DDX19A in gastric cancer were determined using CCK8, plate colony-forming, and Transwell migration assays. The specific mechanism of DDX19A in gastric cancer cells was studied using western blotting, RNA-binding protein immunoprecipitation, mRNA half-life detection, and nuclear and cytoplasmic RNA isolation.

**Results:**

DDX19A was highly expressed in gastric cancer and positively associated with malignant clinicopathological features and poor prognosis. Additionally, DDX19A promoted gastric cancer cell proliferation, migration, and epithelial–mesenchymal transition phenotypes. Mechanistically, DDX19A activated the PI3K/AKT pathway by upregulating phosphatidylinositol-3-kinase (PIK3CA) expression. Furthermore, DDX19A interacted with PIK3CA mRNA, stabilized it, and facilitated its export from the nucleus.

**Conclusions:**

Our study reveals a novel mechanism whereby DDX19A promotes the proliferation and migration of gastric cancer cells by enhancing the stability and nuclear export of PIK3CA mRNA, thereby activating the PI3K/AKT pathway.

## Introduction

Gastric cancer (GC) is one of the most frequent causes of cancer-related deaths worldwide, with over 1 million estimated new cases and approximately 769,000 deaths annually [1]. Owing to the insidious onset and atypical manifestations of early-stage GC, most patients are diagnosed with advanced GC [2]. Despite significant advances in surgery, chemoradiotherapy, targeted therapy, and immunotherapy in recent years, the prognosis of patients with advanced GC remains poor, with a 5-year overall survival below 25% [3]. Therefore, clinical diagnosis and treatment must further identify markers to predict the development of GC and explore the molecular mechanisms underlying GC development.

DEAD-box (DDX) RNA helicase family members are characterized by a generic Asp-Glu-Ala-Asp (D-E-A-D) motif that is involved in all facets of RNA metabolism, including ribosomal biogenesis, RNA nuclear export, translation initiation and termination, and mRNA degradation [4–6]. Emerging evidence suggests that cancer driver events are induced by disturbances in RNA expression or processing [7, 8].

Given the strong regulatory effect of DDXs on RNA, many studies have confirmed that DDX family members are pivotal in tumorigenesis and development [9, 10]. DDX19A, a member of the DDXs family, is located on the human chromosome 16 and comprises 478 amino acid residues [11]. The amino acid sequences of DDX19A and DDX19 protein are 96% identical; thus, DDX19A is annotated as a DDX19-like protein [11, 12]. DDX19 is well known for its intrinsic function in mRNA export from the nucleus to the cytoplasm [13–15].

DDX19A is a novel cytosolic RNA sensor that mediates NLRP3-dependent inflammasome activation during virus infection [16]. To date, the functions of DDX19A in cancer have rarely been reported. Previous research revealed that DDX19A was highly expressed in cervical squamous cell carcinoma and promoted cell metastasis by inducing NOX1-mediated reactive oxygen species production [17], indicating that DDX19A had a cancer-promoting effect. However, another study reported low DDX19A expression in ovarian cancer tissues [18], indicating that DDX19A exerts an antitumor effect. However, the potential function and molecular mechanism of DDX19A in GC progression remains unknown and requires further exploration.

In this study, we aimed to explore the expression pattern, biological function, and possible regulatory mechanism of DDX19A in GC, thereby providing new insights for the diagnosis and therapy of GC.

## Methods

### Tissue specimens

Commercially available GC tissue microarray (TMA) slides (HStmA180Su20 and HStmA160CS01) were purchased from Shanghai Outdo Biotech, Ltd. (Shanghai, China). The TMA chips contained 184 samples of GC tissues and 156 samples of paracancerous tissues, and 104 of these individuals had available data for 4–6 years of follow-up. Twenty-four pairs of fresh GC tissues and adjacent normal gastric mucosa were collected during surgery at the Department of Gastrointestinal Tumor Surgery, Affiliated Hospital of Chengde Medical College. This study was approved by the Ethics Committee of Chengde Medical College.

### Immunohistochemistry (IHC)

IHC was performed using an UltraSensitive™ SP immunohistochemical kit (Maixin, Fuzhou, China), according to the manufacturer’s instructions. Briefly, TMA sections were deparaffinized with xylene and hydrated with gradient alcohol. The sections were treated with citrate antigen retrieval buffer (pH 6.0) in an autoclave for 3 min for antigen repair, and subsequently blocked in goat serum for 30 min at 37℃, and then incubated with anti-DDX19A rabbit polyclonal antibody (CUSABIO, CSB-PA889116HA01HU, Wuhan, China) at 1:100 dilution overnight at 4℃. The sections were washed with phosphate buffer saline and incubated with secondary antibodies using an IHC Kit (KIT9921; MaiXin) the following day. Immunostaining was performed using a DAB kit (DAB-0031, MXB Biotechnologies, China), and the sections were counterstained with hematoxylin for 15 min. The IHC score of DDX19A expression was based on both the DDX19A staining intensity (0, no staining; 1, weak staining; 2, moderate staining; and 3, strong staining) and percentage of DDX19A-stained tumor cells (0, < 5% stained cells; 1, 6–25% stained cells; 2, 26–50% stained cells; 3, 51–75% stained cells; and 4, 76–100% stained cells). The DDX19A staining intensity and DDX19A-stained tumor cell scores were multiplied to obtain a final score ranging from 0 to 12 [19]. Tissues with IHC scores < 6 were considered negative for DDX19A (labeled “-”), and those with IHC scores ≥ 6 were classified as positive for DDX19A (labeled “+”).

### Cell culture

The human gastric mucosal epithelial cell line (GES-1) and human GC cell lines (MGC-803, AGS, BGC-823, and HGC-27) were purchased from the Cell Bank of Type Culture Collection (China Academy of Sciences, Shanghai, China). GES-1, MGC-803, BGC-823, and HGC-27 were cultured in RPMI-1640 medium (Gibco, Carlsbad, CA) with 10% fetal bovine serum (FBS) (Biological Industries, USA) as routine. AGS was maintained in advanced DMEM/F12 medium (Gibco, Carlsbad, CA), supplemented with 10% FBS. All cell lines were cultivated in a thermal incubator at 37 °C under 5% CO_2_.

### Cell transfection

siRNAs for the knockdown of *DDX19A* and scramble siRNA (negative control) were designed and synthesized by Ribobio (Guangzhou, China). The siRNA oligo sequences for DDX19A were: si-DDX19A#1, GCTTCAATCGACCCTCCAA; si-DDX19A#2, TGCCGTTCGAGGCAATAAA. *DDX19A-*Flag overexpression plasmid and control vector PCMV3 were purchased from Sino Biological (Beijing, China). AGS and MGC-803 cells were transfected with plasmid DNA and siRNA using Lipofectamine 3000 reagent (Invitrogen, USA), following the manufacturer’s protocol.

For stable transfection, MGC-803 cells were transfected with the lentivirus sh-DDX19A and negative control (sh-NC) (Hanheng Biotechnology Co. Ltd., Shanghai, China) according to the manufacturer’s instructions, and stable cell lines were obtained through puromycin selection.

### CCK8 and plate colony-forming assays

GC cells transfected with siRNA or overexpression plasmids were seeded into 96-well plates (MGC-803: 1000 cells/well; AGS: 2000 cells/well). After 4–5 h, the cells were incubated with 10% CCK8 reagent (ApexBio, Houston, USA) for 1 h. The absorbance was determined at 450 nm, using a BioTek CYT5M multifunctional microplate reader (BioTek, Winooski, VT, USA), and these measurements were performed continuously for 5 days.

For the plate colony-forming assay, transfected cells were seeded into 6-well plates at 500 cells/well and cultured for 7–14 days on the growth state of the cells. The culture medium was replaced after approximately 3–4 days. After fixing in pre-cooled methanol for 15 min, the colonies were stained with 0.1% crystal violet dye for 20 min, and the number of colonies (≥ 50 cells) were counted.

### Transwell migration assay

For migration assays, 3 × 10^4^ transfected MGC-803 cells or 5 × 10^4^ transfected AGS cells were plated in 200 µL of 2% FBS medium to the upper chambers; 600 µL medium containing 20% FBS was added into the bottom chamber. After incubation for 24 h, the invaded cells were fixed in pre-cooled methanol, stained with 0.1% crystal violet, and counted under a microscope.

### Real-time polymerase chain reaction (PCR)

Total RNA was extracted from tissues and cells using the TRIzol (BS258A, Biosharp, Hefei, China) method and then reverse transcribed to cDNA using the Quantscript RT Kit (RK20429, Abclonal, Wuhan, China). We used 2X Universal SYBR Green Fast qPCR Mix (PK21203, Abclonal, Wuhan, China) for quantitative PCR (qPCR) analysis, and gene expression was detected using a thermocycler (CFX96 Touch, BIO-RAD, CA, USA). The qPCR results were calculated with a 2^−ΔΔCt^ method using GAPDH as an internal control. The primer sequences were: DDX19A, 5′-CATGGGCTTCAATCGACCCT-3’ and 5′-GCACAGACACTGGGGGTATC-3′; PIK3CA, 5′-CCACGACCATCATCAGGTGAA-3′ and 5′-CCTCACGGAGGCATTCTAGGGT-3′; GAPDH, 5′-GCACCGTCAAGGCTGAGAAC-3′ and 5′-TGGTGAAGCGCCAGTGGA-3′.

### Western blotting

Total protein was extracted from cells and tissues using RIPA lysis buffer (R0010, Solaibao, Beijing, China) containing 1% protease inhibitor (P6731, Solaibao, Beijing, China) and phosphatase inhibitors (P1260, Solaibao, Beijing, China), and protein quantitation was performed using the BCA assay. Protein lysate, 30 µg/well was separated by 10% SDS-PAGE and then transferred onto a PVDF membrane (Millipore, Burlington, MA, USA) by wet transfer method. After blocking in 5% skimmed milk for 2 h at 37℃, the membranes were incubated with the following primary antibodies at 4 °C overnight: the anti-DDX19A (CSB-PA889116HA01HU, Cusabio, Wuhan, China, diluted 1:2000), anti-PIK3CA (67071-1-Ig, Proteintech, Wuhan, China, diluted 1:2000), anti-PI3Kp85α (60225-1-Ig, Proteintech, Wuhan, China, diluted 1:2000), anti-AKT (A18120, Abclonal, Wuhan, China), anti-p-AKT (ser473) (AF0016, Affinity Changzhou, China, diluted 1:1000), anti-GAPDH (60004-1-Ig, Proteintech, Wuhan, China, diluted 1:10000). On the second day, the membranes were hybridized with HRP-conjugated AffiniPure Goat anti-rabbit/ mouse IgG (RS0001/RS0001, ImmunoWay, TX, USA, diluted 1:10000) for 1 h at 37℃. The protein bands were visualized using an enhanced chemiluminescence kit (BL523A; Biosharp, Hefei, China), and the detection results were quantified using ImageJ (NIH, Bethesda, MD, USA).

### RNA-binding protein immunoprecipitation (RIP)

The RIP assay for DDX19A and DDX19A-binding RNA was performed using an RNA Immunoprecipitation Kit (P0102, Geneseed, Guangzhou, China), following the manufacturer’s protocol. AGS cells transfected with the DDX19A-Flag plasmid for 48 h were harvested and lysed using RIP lysis buffer. After resuspension and washing, 50 µL magnetic beads were incubated with 5 µg anti-Flag (20543-1-AP, Proteintech, Wuhan, China) or anti-IgG (AC011, Abclonal, Wuhan, China) antibody overnight at 4 °C. For immunoprecipitation, after centrifugation, the supernatant was incubated in the magnetic beads-antibody complex for 6–8 h at 4 °C. The immunoprecipitate was centrifuged and prepared for western blotting and reverse transcription qPCR (RT-qPCR) analyses. The PCR data were processed as follows: ΔΔct = (Ct IP - Ct Input) - (Ct IgG - Ct input). Gene expression levels were represented as gene expression levels in treatment groups relative to the control group using the 2^−ΔΔCt^ method.

### mRNA half-life detection

AGS and MGC-803 cells were seeded in 6-well plates. After up to 80% confluency in 16–18 h, cells were treated with 5 µg/mL actinomycin D (GC16866, Glpbio, Montclair, USA) or DMSO (D8371, Solaibao, Beijing, China) and collected after 0, 1, 2, 3, and 4 h of treatment. Total RNA was extracted using TRIzol reagent, and RNA expression levels were analyzed using RT-qPCR.

### Nuclear and cytoplasmic RNA isolation

Nuclear and cytoplasmic RNA extraction was performed using the Cytoplasmic & Nuclear RNA Purification kit (21,000, Norgen, Thorold, ON, Canada), following the manufacturer’s instructions. Briefly, AGS was lysed in 200 µL Lysis Buffer J on ice for 5 min. After centrifugation at 14,000 rpm for 5 min at 4 °C, the supernatant was collected for cytoplasmic RNA, and the precipitate contained nuclear RNA. RNA abundance in the cytoplasmic and nuclear fractions was detected using RT-qPCR and normalized to ACTIN and U1, respectively.

### Xenograft mouse model

Six-week-old female BALB/c nude mice were purchased from Weitong Lihua Experimental Animal Technology Co. Ltd. (Beijing, China). MGC-803 cells (5 × 10^6^) stably transfected with sh-DDX19A or sh-NC were subcutaneously injected into the right armpit of mice (*n* = 5, random allocation). The tumor length (L) and width (W) were measured every 3 days, and the subcutaneous tumor volume (mm^3^) was calculated using the formula V = (L × W^2^)/2. Twenty-one days after the injection, mice were sacrificed, and tumor tissues were weighed and sampled for subsequent experiments. All experimental procedures involving animals were approved by the Experimental Animal Ethics Committee of Chengde Medical College.

### Statistical analysis

SPSS 20.0 (IBM, Chicago, IL, USA) was used for statistical analyses and GraphPad Prism 7.0 (GraphPad Software Inc. San Diego, CA, USA) for plotting data. Differences in DDX19A expression between cancer tissues and adjacent tissues, and the association between DDX19A expression and clinical pathological parameters were analyzed using the chi-square test. Differences between two groups were determined using the Student’s *t* test. *P* < 0.05 was considered statistically significant.

## Results

### DDX19A is highly expressed in human GC and associated with poor prognosis

**Fig. 1:**
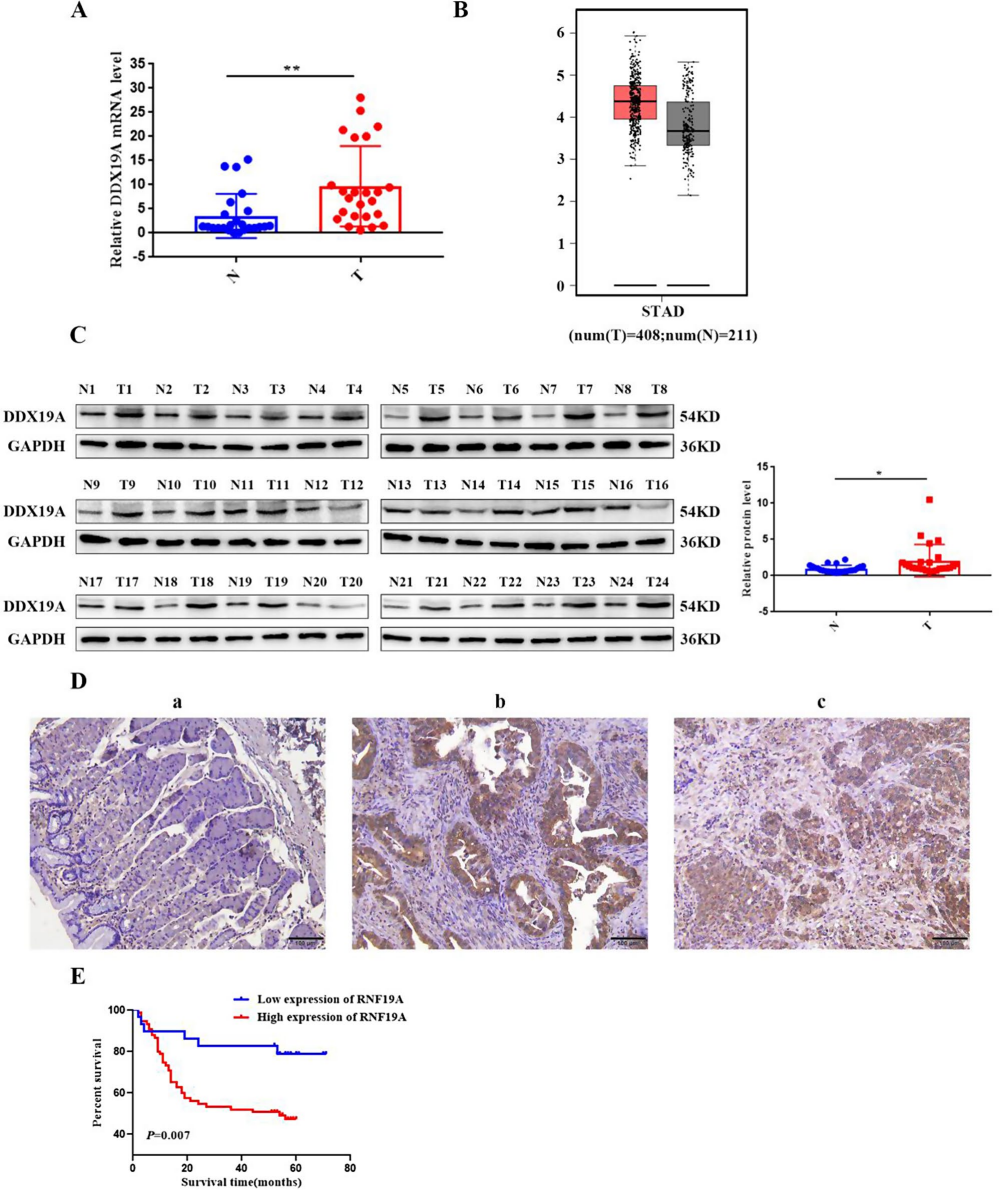
DDX19A expression is upregulated in GC, and high DDX19A expression is associated with poor prognosis. (**A**) DDX19A mRNA levels as detected using RT-qPCR in 24 paired fresh frozen GC tissues. (**B**) DDX19A expression level in 408 GC samples and 211 normal controls from the GEPIA database. (**C**) DDX19A protein levels as detected by western blotting in 24 paired fresh frozen GC tissues. (**D**) Immunohistochemical results of DDX19A (200×). (**a**) Normal gastric tissue; (**b**) high-medium differentiated GC tissue; (**c**) poorly differentiated GC tissue. (**E**) Kaplan–Meier curves for overall survival analysis by DDX19A expression in patients with GC. *p < 0.05, **p < 0.01. GC, gastric cancer; STAD, stomach adenocarcinoma

To evaluate the expression of DDX19A in GC samples, we performed RT-qPCR and western blotting on fresh tissues and IHC on paraffin-embedded tissues. The mRNA expression of DDX19A in GC tissues was measured using RT-qPCR. DDX19A was highly expressed in GC tissues when compared with paracancerous tissues (Fig. 1A). The overexpression of DDX19A mRNA in GC tissues was confirmed using the GEPIA database [20] (Fig. 1B). Western blotting analysis confirmed a significant increase in DDX19A protein expression in GC tissues (Fig. 1C). DDX19A in 184 GC tissues and 156 adjacent non-cancer tissues were detected by IHC, and DDX19A was significantly upregulated in GC tissues when compared to adjacent tissues (Fig. 1D; Table 1). DDX19A expression positively correlated with tumor grade, depth of invasion, lymph node metastasis, and TNM stage, but not with other clinicopathological features (patient age and sex, tumor size) (Table 2). Kaplan − Meier analysis of 104 GC tissues revealed that higher expression of DDX19A suggested poorer overall survival (Fig. 1E).

**Table 1:** DDX19A expression in para-carcinoma and gastric cancer tissues

Group	n	DDX19A		*χ* ^*2*^	*P*
		Low	High		
Para-carcinoma	156	95	61	46.285	0.000
Gastric carcinoma	184	45	139		

**Table 2:** Association of DDX19A expression with clinicopathological characteristics of GC

Group	*n*	DDX19A		χ^2^	*P*
		Low	High		
Gender					
Male	136	33	103	0.010	0.919
Female	48	12	36		
Age					
≤ 60	53	10	43	1.258	0.262
> 60	131	35	96		
Grade					
I–II	62	21	41	4.486	0.034
III	122	24	98		
Lymph node metastasis					
No	42	24	18	31.472	0.000
Yes	142	21	121		
TNM stage					
I + II	67	28	39	17.137	0.000
III + IV	117	17	100		
Depth of invasion					
≤Serous layer	25	15	10	19.783	0.000
> Serous layer	159	30	129		
Tumor size					
≤ 4 cm	100	28	72	1.489	0.222
> 4 cm	84	17	67		

### DDX19A enhances the proliferation and migration of GC cells

**Fig. 2:**
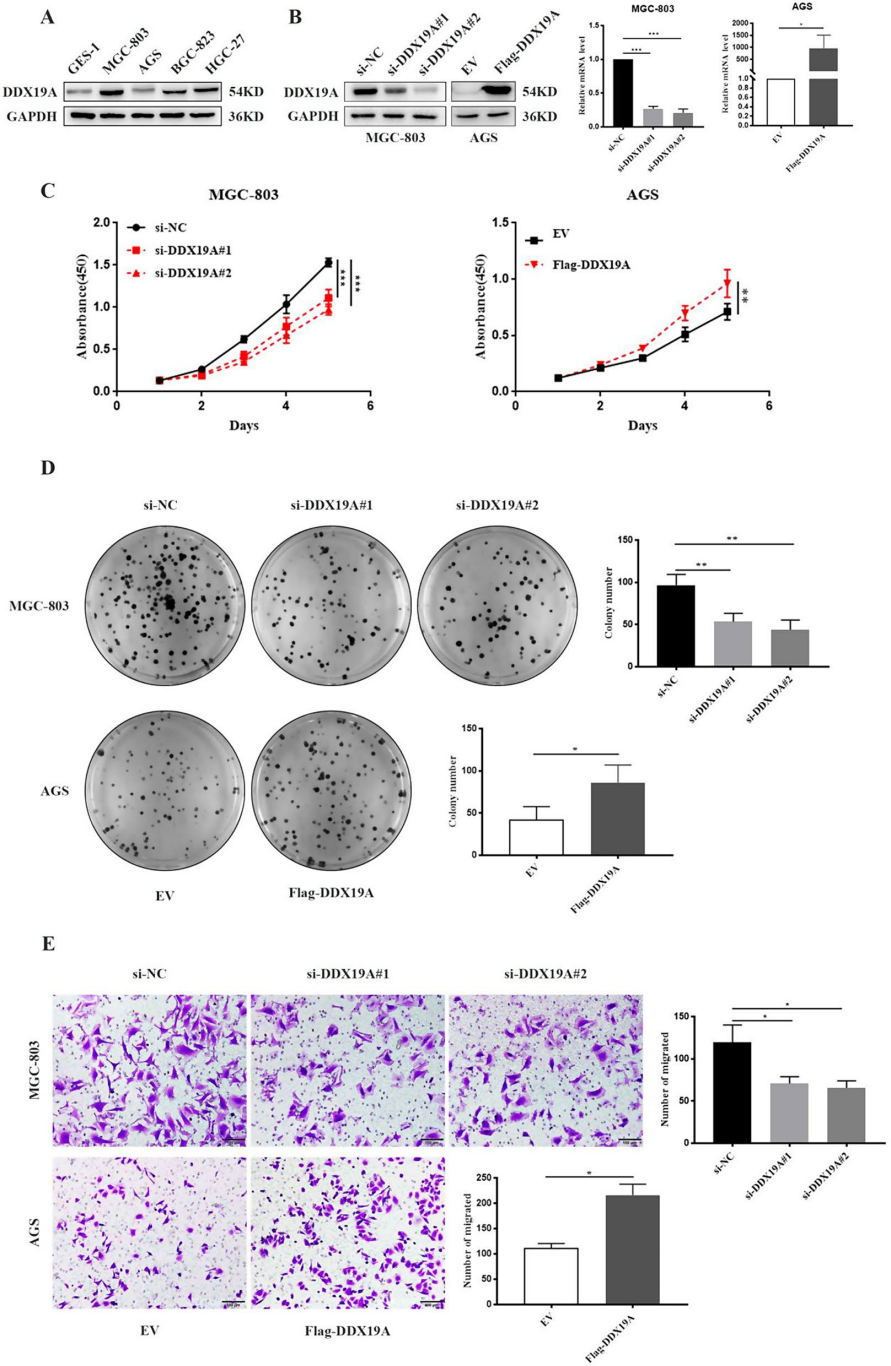
DDX19A induces proliferation and migration of GC cells. (**A**) Analysis of DDX19A expression levels in GC cell lines and a normal gastric epithelium cell line (GES-1) by western blotting. (**B**) Silencing and overexpression efficiency assessed using western blotting and RT-qPCR analyses. (**C**) Growth curves of MGC-803 and AGS cells after transfection with DDX19A siRNA or DDX19A-overexpressing plasmid assessed using CCK8 assays. (**D**) Colony formation after depletion or overexpression of DDX19A assessed through plate cloning experiments. (**E**) GC cell migration assessed through Transwell migration assay. *p < 0.05, **p < 0.01, ***p < 0.001

To investigate the biological role of DDX19A in GC, we evaluated its expression in various GC cell lines and normal human gastric mucosal epithelial cells. DDX19A levels were induced in GC cell lines (MGC-803, AGS, BGC-823, and HGC-27) and compared to those in GES-1 cells (Fig. 2A). AGS and MGC-803 cells had low and high levels of DDX19A, respectively; hence, these two cell lines were selected for subsequent studies. Next, we confirmed the overexpression and knockdown efficiency of Flag-DDX19A and si-DDX19A using western blotting and RT-qPCR (Fig. 2B). To detect the possible effects of DDX19A on GC cell growth and migration, CCK8, colony formation, and Transwell assays were performed. Enforced overexpression of DDX19A markedly enhanced the cell viability, colony-forming capability, and migration of human GC cells. In contrast, DDX19A knockdown significantly suppressed cell growth and migration (Fig. 2C–E).

### DDX19A knockdown inhibits GC cell growth *in vivo*

**Fig. 3:**
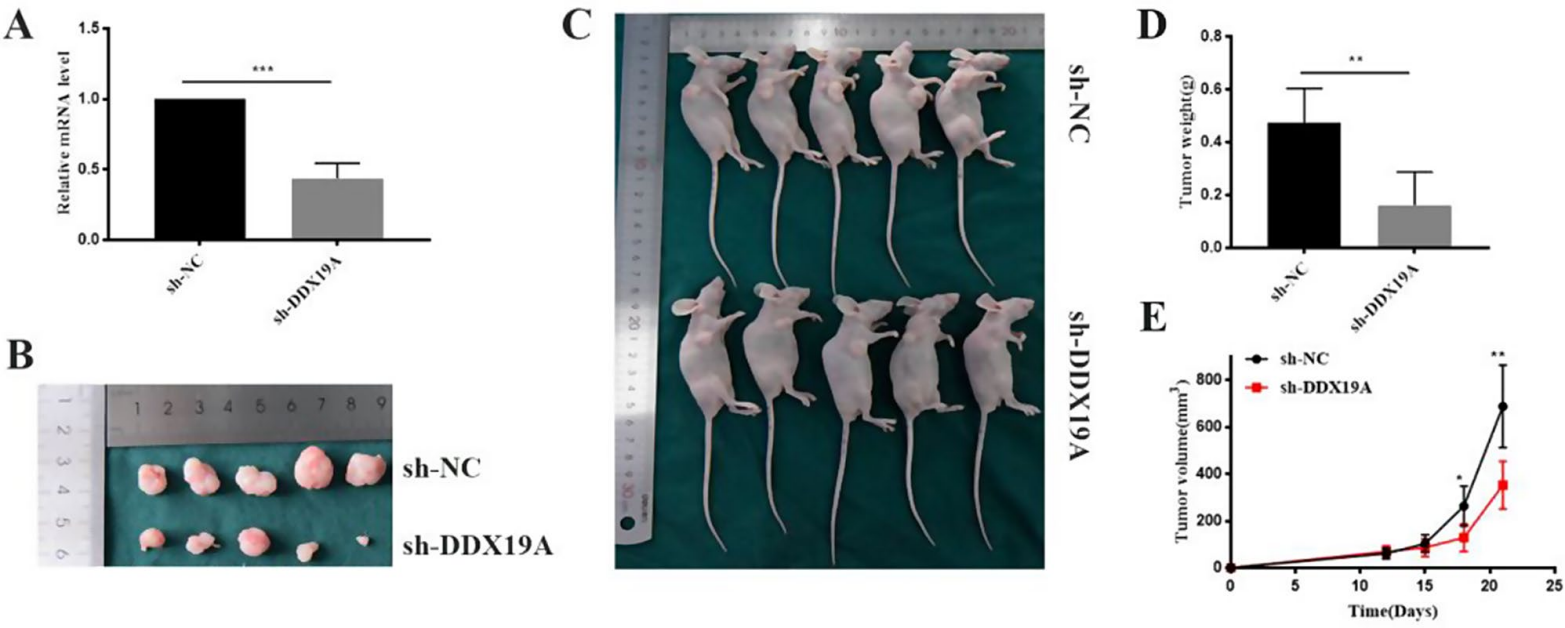
DDX19A knockdown inhibits tumor growth *in vivo*. (**A**) Efficiency of *DDX19A* knockdown. (**B**) Images of tumors removed from mice from the sh-DDX19A and sh-NC groups. (**C**) Images of nude mice from the sh-DDX19A and sh-NC groups. (**D**) Average tumor weight of xenografts. (**E**) Tumor growth curves.*p < 0.05, **p < 0.01, ***p < 0.001

To determine whether DDX19A affects GC cell growth in vivo, we established a tumor xenograft model by subcutaneously injecting mice with *DDX19A* knockdown MGC-803 cells. The efficiency of the *DDX19A* knockdown was confirmed using RT-qPCR (Fig. 3A). Compared with those in the control groups, the tumor volume and weight in the *DDX19A* knockdown group were significantly decreased (Fig. 3B–E).

### DDX19A promotes epithelial–mesenchymal transition (EMT) in GC cells

**Fig. 4:**
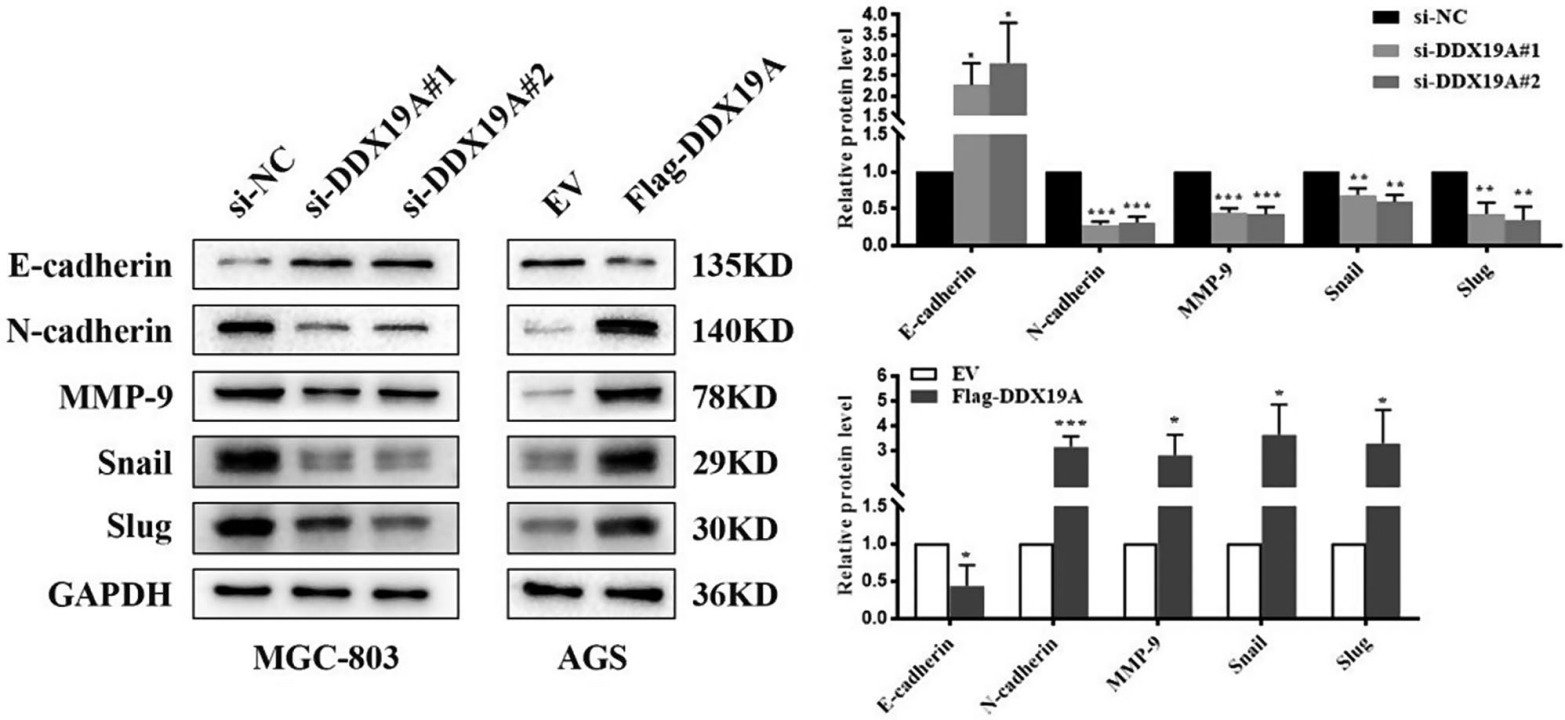
DDX19A regulates EMT markers in GC cells. *p < 0.05, **p < 0.01, ***p < 0.001

The EMT program is a key process associated with the migration and invasion of GC [21]. We examined the effect of DDX19A on EMT in GC cells. Western blotting results revealed that the overexpression of DDX19A increased the expression levels of N-cadherin, MMP-9, Snail, and Slug, but reduced E-cadherin expression. In contrast, after the knockdown of DDX19A in MGC-803 cells, EMT-related proteins exhibited the opposite trend (Fig. 4). These findings indicate that DDX19A may be associated with the EMT phenotype of GC cells.

### DDX19A activates the PI3K/AKT signaling pathway

**Fig. 5:**
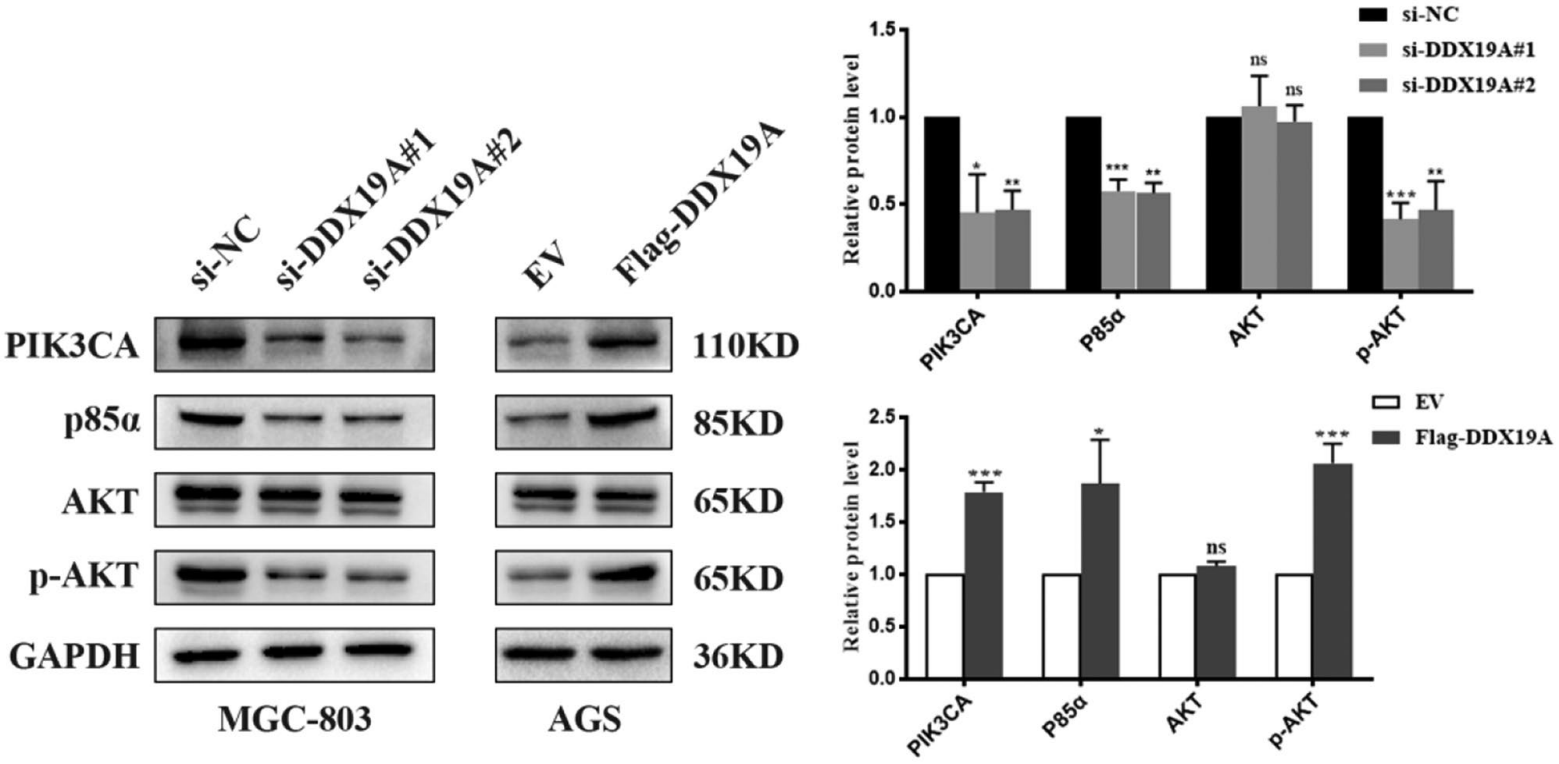
DDX19A activates PI3K/AKT signaling pathway. *p < 0.05, **p < 0.01, ***p < 0.001, ^ns^not statistically significant

To further explore the mechanism underlying DDX19A in GC, we examined the classic PI3K/AKT signaling pathway, which is closely associated with cancer. Western blotting analysis revealed that DDX19A upregulated the protein expression of PIK3CA, p85α, and p-AKT (phosphorylated AKT), but did not affect AKT expression. In contrast, DDX19A knockdown had the opposite effects (Fig. 5). These findings indicate that DDX19A may activate the PI3K/AKT pathway in GC cells.

### PIK3CA inhibitor MLN1117 attenuates the promotion effect of DDX19A overexpression on GC cells

**Fig. 6:**
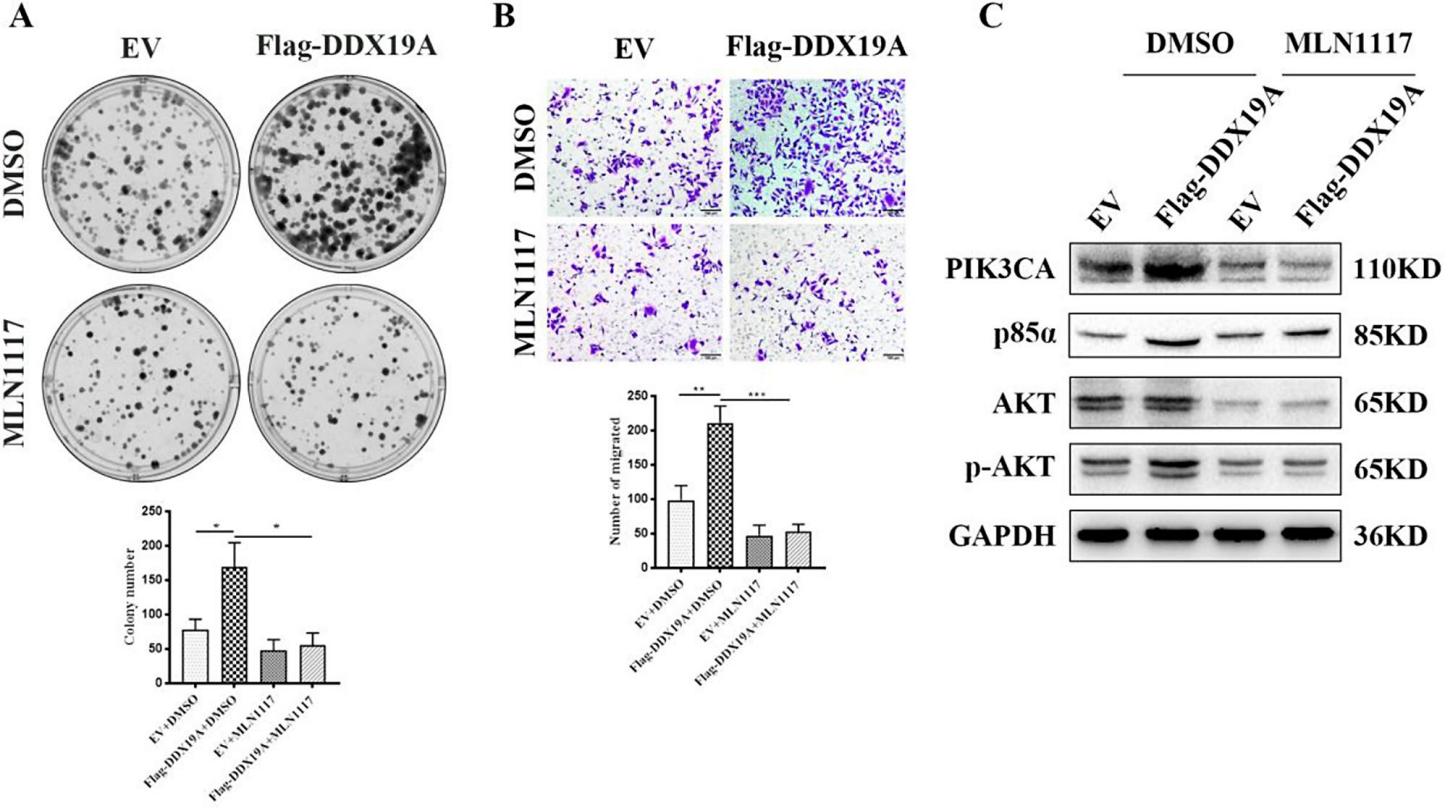
PIK3CA inhibitor MLN1117 attenuates the promotion effect of DDX19A overexpression on GC cells. (**A**) Colony formation and (**B**) migration assays with AGS cells transfected with a DDX19A-overexpressing plasmid and incubated with the PIK3CA inhibitor MLN1117 (10 µM). (**C**) Expression of proteins involved in PI3K/AKT signaling in AGS cells transfected with a DDX19A-overexpressing plasmid and treated with DMSO or the PIK3CA inhibitor MLN1117 (10 µM). *p < 0.05, **p < 0.01, ***p < 0.001

PI3K-PIK3CA is located upstream of PI3K/AKT and is critical for activating the signaling pathway; thus, we speculated that DDX19A might modulate the PI3K/AKT pathway by relying on PIK3CA. To confirm this hypothesis, we designed a rescue experiment by adding the PIK3CA-specific inhibitor MLN1117 to DDX19A-overexpressed cells. As illustrated in Fig. 6A and B, MLN1117 treatment reversed DDX19A-mediated growth enhancement in AGS cells. Additionally, treatment with MLN1117 rescued the effects of DDX19A on the expression of PIK3CA downstream proteins (Fig. 6C). These findings demonstrated that DDX19A activates the PI3K/AKT pathway by regulating PIK3CA expression.

### DDX19A promotes nuclear export and stabilizes PIK3CA mRNA through binding to PIK3CA

**Fig. 7:**
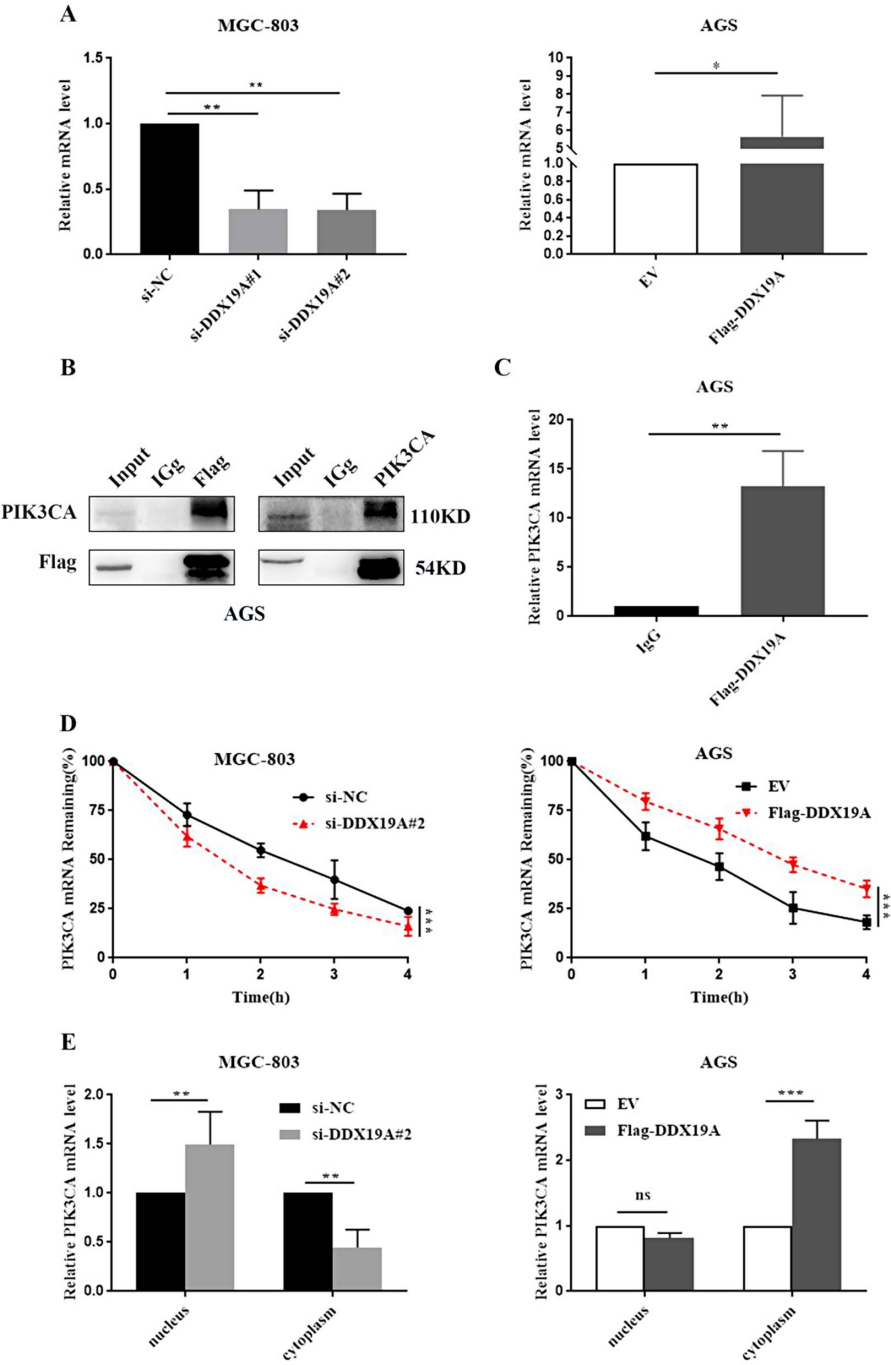
DDX19A promotes nuclear export and stabilizes PIK3CA mRNA. (**A**) Effect of DDX19A on PIK3CA mRNA expression. (**B**, **C**) Interaction between DDX19A and PIK3CA in AGS cells overexpressing Flag-DDX19A assessed using RIP assay, western blotting, and RT-qPCR. (**D**) Effects of *DDX19A* knockdown (left) and overexpression (right) on PIK3CA mRNA stability at the indicated time points measured using RT-qPCR. (**E**) PIK3CA mRNA levels in the nuclear and cytoplasmic fractions of *DDX19A* knockdown MGC-803 cells (left) and *DDX19A* overexpression AGS cells (right) assayed using RT-qPCR. *p < 0.05, **p < 0.01, ***p < 0.001, ^ns^not statistically significant

*DDX19*, a gene that is most similar to DDX19A, plays a critical role in mRNA export from the nucleus to the cytoplasm. We investigated whether DDX19A exported PIK3CA mRNA. First, RT-qPCR was performed to determine whether DDX19A regulates PIK3CA at the transcriptional level. As illustrated in Fig. 7A, PIK3CA mRNA decreased in MGC-803 cells with DDX19A knockdown and significantly increased in AGS cells overexpressing DDX19A. Next, we performed RIP assays in AGS cells and discovered that the PIK3CA protein/mRNA co-precipitated with DDX19A, indicating a specific interaction between the DDX19A protein and PIK3CA protein/mRNA (Fig. 7B and C). To further determine whether DDX19A maintains PIK3CA mRNA stabilization, we assayed mRNA half-life by treating GC cells with actinomycin D. DDX19A depletion dramatically decreased the half-life of PIK3CA mRNA, whereas DDX19A overexpression increased its half-life (Fig. 7D), suggesting that DDX19A enhanced the stability of PIK3CA mRNA. Furthermore, nuclear and cytoplasmic fractionation assays revealed that the proportion of cytoplasmic PIK3CA mRNA increased in DDX19A-upregulated GC cells (Fig. 7E), supporting the finding that DDX19A enhanced the export of PIK3CA mRNA. These data imply that DDX19A stabilizes and enhances PIK3CA mRNA export by binding to PIK3CA.

## Discussion

DDX19A is a tumor promoter in cervical cancer; however, its clinical significance and function in GC remain unclear. This study is the first to demonstrate that DDX19A is upregulated in GC tissues and that the overexpression of DDX19A is correlated with a range of malignant clinicopathological features and poor prognosis of GC. In vitro and in vivo experiments confirmed that DDX19A enhanced the proliferation and migration of GC cells. Moreover, DDX19A contributes to the EMT tumorigenic phenotype of GC cells. EMT is a multistep biological process wherein epithelial cells are transformed into mesenchymal cells [22]. EMT is involved in the migration and invasion of various malignant tumors, and EMT activation in tumor cells may promote cancer cell progression and metastasis [23, 24]. Our findings indicate that DDX19A may act as an oncogene that promotes GC progression. Consistent with our observations, previous studies have revealed that several DDXs have multiple functions in various biological processes during tumorigenesis [9, 10, 25–28].

We investigated the molecular mechanism through which DDX19A acts as a tumor driver in GC and identified the PI3K/AKT pathway as a downstream target of DDX19A in GC. Our results revealed that DDX19A activates this signaling pathway.

The PI3K/AKT signaling pathway plays an important role in tumorigenesis by regulating cell cycle progression, cell proliferation, and migration [29, 30]. Previous studies have indicated that other DDX members, such as DDX51, DDX5, DDX11, and DDX54, are critical for regulating PI3K/AKT signaling [31–34]. Notably, all four compounds exert this effect on human cancers.

Mechanistically, DDX19A interacted with and efficiently facilitated the nuclear export of PIK3CA mRNA, thereby maintaining its stability. Subsequently, increased expression of PIK3CA activated the PI3K/AKT signaling pathway to promote the malignant function of GC cells. Accordingly, our data indicated that treatment with a PIK3CA inhibitor restored the phenotypes induced by DDX19A overexpression. PI3K is a heterodimeric lipid kinase composed of a regulatory subunit (p85α) and a catalytic subunit (p110α), which plays a crucial role in cancer development [35]. The class I PIK3CA catalytic fractional unit, encoded by the PIK3CA gene located on chromosome 3 at 3q26.3, catalyzes the phosphorylation of PIP2 to form PIP3, which acts as a recruitment docking site for AKT [36–39]. Previous studies have revealed that abnormally elevated PIK3CA contributes to the growth and invasion of multiple cancers, including ovarian, breast, colorectal, and gastric cancers [40, 41]. Previous data has identified PIK3CA as the miR-199a-3p downstream target, which is negatively regulated by the latter [42]. This suggests that PIK3CA is subject to post-transcriptional regulation. In this study, we discovered that DDX19A binds to PIK3CA and facilitates its nuclear export. However, the structural basis of the binding between DDX19A and PIK3CA remains unclear. This key issue requires further investigation.

## Conclusions

This study suggests that DDX19A acts as an oncogenic factor in GC by activating cell proliferation and migration via the PI3K/AKT signaling pathway. The specific regulatory mechanism involves promoting PIK3CA mRNA nuclear export. In conclusion, we identified DDX19A as a novel oncogenic biomarker and promising therapeutic target in patients with GC, which may contribute to exploring the biological function of DDX19A in cancer.

## Data Availability

The data that support the fndings of this study are available from the corresponding author upon reasonable request.

## Abbreviations

DDX: DEAD-box
EMT: Epithelial–mesenchymal transition
FBS: Fetal bovine serum
GC: Gastric cancer
GES-1: Gastric mucosal epithelial cell line
IHC: Immunohistochemistry
DAB: 3,3’-diaminobenzidine
CCK8: Cell Counting Kit-8
DMSO: Dimethyl sulfoxide
TNM: Tumor node metastasis classification
PIK3CA: Phosphatidylinositol-3-kinase
AKT: Protein kinase B
RIP: RNA-binding protein immunoprecipitation
siRNA: Small interfering RNA
TMA: Tissue microarray
